# Balancing traditional activities and cognitive pharmaceutical services by community pharmacists: a work sampling study

**DOI:** 10.1007/s11096-019-00852-0

**Published:** 2019-05-28

**Authors:** Jeroen van de Pol, Ellen Koster, Anke Hövels, Marcel Bouvy

**Affiliations:** Universiteit Utrecht Faculteit Betawetenschappen, Utrecht, Netherlands

**Keywords:** Cognitive pharmaceutical services, Community pharmacy, Pharmacy research, The Netherlands, Time utilization, Work sampling

## Abstract

*Background* Community pharmacy is undergoing a transition, shifting focus from traditional roles to the provision of cognitive pharmaceutical services. However, traditional activities performed by community pharmacists reduce the amount of available time for implementing and providing such services. Therefore, hampering the community pharmacist in the transition. *Objective* The aim of this study was to identify characteristics of community pharmacists that spend a higher proportion of their time on cognitive pharmacy services and to identify activities that compete with time spent on such activities by community pharmacists. *Setting* Daily community pharmacy practice. *Method* Self-reporting work sampling using smartphone technology was used to register the activities of community pharmacists. Participating pharmacists recorded their current activity five times per working day for 6 weeks and also completed an online survey about baseline characteristics. *Main outcome measure* Time utilization. *Results* Ninety-one Dutch community pharmacists provided work-sampling data. The results showed that Dutch community pharmacists are predominantly spending less time on managerial activities when spending more time on cognitive services (from 25.7% to 14.5%, *p* = 0.016). Pharmacists who are spending more time on such services, want to spend even more time on direct patient contact compared to pharmacists who spend less time on it (*p* = 0.030). *Conclusion* This study shows that community pharmacists that spend more time on cognitive pharmacy services are devoting less time on managerial activities, logistics and other activities. Pharmacists spending more time on cognitive pharmaceutical services are mostly locum pharmacists or work at a pharmacy located in a residential area with largely older inhabitants.

## Impacts on Practice

- Activities, predominantly belonging to pharmacy management and logistical processes, seem plausible candidates for task delegation.
- Delegating quality assurance activities can save large amounts of time for community pharmacists in the Netherlands, but is hampered by legislation.
- Delegating time consuming activities to supporting staff members will enable pharmacists to spend more time on cognetive pharmaceutical services.

## Introduction

Community pharmacy around the world is undergoing a transition. Traditional pharmacist tasks such as the compounding and distribution of medicines gradually become less prominent and are being replaced by cognitive pharmaceutical services (CPS). This transition, like in other countries, is also ongoing in the Netherlands.

CPS has been defined as “the use of specialized knowledge by the pharmacist for the patient or health professionals for the purpose of promoting effective and safe drug therapy” [1]. Examples of CPS are clinical medication review (CMR), discharge counseling or Inhaler Technique Assessment Service (ITAS) [2].

The urgency of this transition was already emphasized in the 90’s of the previous century, as it was expected that solely dispensing was not going to be a sustainable basis for pharmacy practice [3]. It has also been stated earlier that the sole dispensing of medicines cannot be seen as pharmaceutical care [4]. However, over two decades later, the transition is still unfinished.

In the meantime, policymakers are confronted with an ageing population and the introduction of more complex medication use by patients. In this increasingly demanding healthcare setting policymakers and insurance companies are scrutinizing all healthcare professionals and expecting them to provide effective and efficient healthcare.

This increases the need of a more clinical role of pharmacists to support patients with multimorbidity and polypharmacy. It has been found in previous research that community pharmacist have a positive impact on the healthcare system [5] and that pharmacist led interventions can benefit patients with diverse conditions such as high blood pressure, hyperlipidemia and tobacco dependence [6, 7]. Also, numerous studies have shown that CMR performed by pharmacists, identifies and solves drug related problems (DRP) and inappropriate prescribing [8, 9] and improves adherence to medication [10].

Recent research has shown that community pharmacists in the Netherlands have a diverse set of daily recurring activities that are all competing over the available time of the pharmacist [11]. These daily recurring activities are often essential to manage the pharmacy, but may hamper the community pharmacist in the amount of time he/she can dedicate to CPS [4, 12–18].

### Aim of the study

The aim of this study was to identify characteristics of community pharmacists that spend a higher proportion of their time on CPS and to identify activities that compete with time spent on CPS by community pharmacists.

### Ethics approval

The research proposal was approved by the Institutional Review Board (IRB) of Utrecht University. The study used a smartphone application called FarmaCheck [11] which provided each participant with a unique *user code* and could not be linked to individual participants. Data was anonymous and treated as confidential.

## Method

### Study design

A cross-sectional study design was used with a work-sampling technique based on self-report of activities at random intervals as described in an earlier paper [11]. In short, participants were provided with a smartphone application to register activities during daily community pharmacy practice.

### Participants

Practicing community pharmacists were recruited through the Utrecht Pharmacy Practice network for Education and Research (UPPER) [11, 19].

### Data collection

During the study period (from January till July 2016), participating community pharmacists registered their daily activities using a smartphone application called ‘FarmaCheck’. This application asked participants at five random times per workday to register their current activity. Pharmacists were asked to do this during six consecutive weeks [11]. To get insights in the background characteristics of participants, each pharmacist was asked to fill in a brief online survey.

### Data analysis

#### Outcome measures

Proportions of time spent on different activities was obtained by dividing the number of times a certain activity was chosen in the smartphone application by the total number of registered activities. Activities were categorized into five activity groups. These five activity groups were comprised of similar activities that were found in previous research [11]. The groups are cognitive pharmaceutical services (CPS), pharmacy management (PM), quality assurance (QA), logistical processes (LP) and other (see Table 1).

**Table 1:** Activity groups. Detailed information regarding each activity group can be found in [11] and “Appendix”

Composed activity groups	Activity groups from [11]
Cognitive pharmaceutical services	Cognitive pharmaceutical services
Pharmacy management	Organizational activities
	Human resource management
	Finance
Quality assurance	Quality assurance
	Final check of prescription
	Clinical risk management
Logistical processes	Logistics
	Dispensing process
Other	Household chores
	Education
	Non-professional encounters and other
	Rest

#### Statistical analysis

Participants who did not complete the online survey or responded to less than 30% of generated alerts were excluded from analysis. Results are presented as the average percentage of alerts that were recorded into one of the five activity groups. Three groups of participants were formed based on the amount of time spent on CPS.

Descriptive statistics were calculated for each group based on background information. Univariate analysis was conducted on the characteristics and the amount of time spent on the five activity groups. Due to the non-parametric distribution of the data, Mann–Whitney *U* tests were performed in the univariate analysis when dealing with dichotomous variables. When dealing with more than two variables, Kruskal–Wallis tests were performed. Statistical analysis were performed using Microsoft Excel and SPSS 23.0.

## Results

In total 11,918 activities were registered by 156 participants. A total of 65 community pharmacists did not provide data on background characteristics and were therefore excluded. The remaining 91 pharmacists registered an activity for 72.4% on average of the alerts. A total of 734 activities coded by the participants as “not at work” were excluded (Tables 2).

**Table 2:** Demographic data. When type of pharmacist is defined as resident, this means that he/she is the pharmacist within the community pharmacy that holds final responsibility for all activities within the community pharmacy practice

Characteristic	*N* = 91
Age in years (mean ± SD)	39.4 ± 10.7
Male gender	30 (33.0%)
Graduation year (mean ± SD)	2002 ± 10
Type of pharmacist:	
Resident and (partial) owner	27 (29.7%)
Resident in paid employment	33 (36.3%)
Locum	31 (34.1%)
Working hours per week (mean ± SD)	36.7 ± 7.32
Pharmacy part of pharmacy chain or partnership	
No	32 (35.2%)
Partnership with < 5 pharmacies	20 (22.0%)
Between 5 and 25 pharmacies	23 (25.3%)
Chain > 25 pharmacies	16 (17.6%)
Community pharmacists stating being in control of time utilization	
Yes, fully in control	48 (52.7%)
More or less in control	43 (47.3%)

**Fig. 1:**
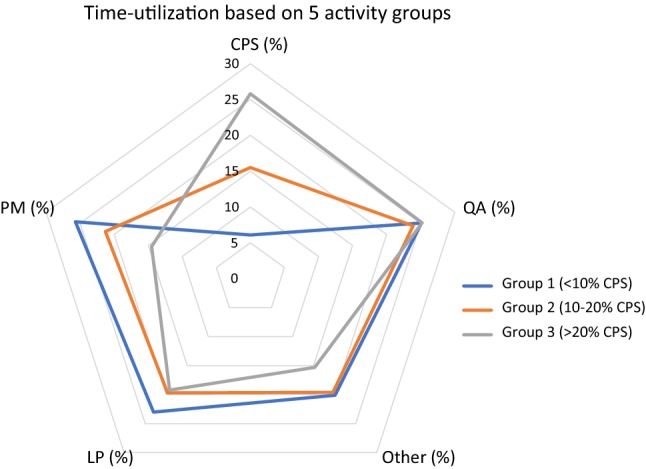
Time utilization based on 5 activity groups

Descriptive analysis of the distribution of the amount of time spent on CPS showed that three groups of approximately equal size could be defined based on the amount of time they spend on CPS (Fig. 1 and Table 3).

**Table 3:** Definition of the three groups based on the amount of time spent on cognitive pharmaceutical services (CPS)

Group	Defined by time spent on CPS (%)	Average time ± SD spent on CPS (%)
1 (*n* = 34)	0–10	6.1 ± 2.8
2 (*n* = 37)	10–20	15.5 ± 2.8
3 (*n* = 20)	> 20	25.8 ± 5.4

Analysis of the amount of time the three groups (based on the amount of time spent on CPS), spent on the 5 different activity groups using the Kruskal–Wallis test provided the results in Table 4.

**Table 4:** Time utilization of pharmacists spending different amounts of time on CPS

	CPS %	PM %	QA %	LP %	Other %
Group 1	6.1	25.7	25.1	23.0	20.1
Group 2	15.5	21.3	23.9	19.7	19.6
Group 3	25.8	14.5	25.2	19.2	15.3
*p* value	< 0.001	0.016	0.874	0.378	0.023

Results acquired using the Kruskal–Wallis test

*CPS* cognitive pharmaceutical services, *PM* pharmacy management, *QA* quality assurance, *LP* logistical processes

## Discussion

The results show significant differences between community pharmacists regarding the amount of time they spend on CPS and can be divided into three groups. Pharmacists that spend more time on CPS, are especially spending less time on pharmacy management, logistics and other activities (Table 5).

**Table 5:** Background information on the three groups. When type of pharmacist is defined as resident, this means that he/she is the pharmacist within the community pharmacy that holds final responsibility for all activities within the community pharmacy practice

	Group 1	Group 2	Group 3	*p* value
Background information	*N* = 34	*N* = 37	*N* = 20	
Gender (%)				0.711
Male	38.2	29.7	30.0	
Age in years (average ± SD)	39.6 ± 10.7	40.7 ± 10.4	36.6 ± 11.2	0.359
Graduation year (average ± SD)	2001 ± 10.0	2000 ± 10.0	2004 ± 10.0	0.312
Type of pharmacist (%)				0.318
Resident and (partial) owner	26.5	40.5	15.0	
Resident in paid employment	41.2	29.7	40.0	
Locum pharmacist	32.4	29.7	45.0	
Working hours per week (average ± SD)	36.3 ± 9.1	36.8 ± 6.1	37.3 ± 6.2	0.470
Self-reported extent of control over time utilization (%)				0.894
Yes, fully in control	55.9	51.4	50.0	
More or less in control	44.1	48.6	50.0	
Pharmacy part of a chain or partnership (%)				0.883
Not part of a chain- or partnership	38.2	32.4	35.0	
Yes. Less than 5 pharmacies	23.5	21.6	20.0	
Yes. Between 5 and 25 pharmacies	17.6	32.4	25.0	
Yes. With over 25 pharmacies	20.6	13.5	20.0	
Age of population of residential area (%)				0.233
Mostly younger inhabitant	11.8	16.2	5.0	
Both young and old inhabitants	52.9	37.8	30.0	
Mostly older inhabitants	35.3	45.9	65.0	
Utilizing centralized prescription processing (central fill) (%)				0.664
Yes	55.9	62.2	50.0	
No	44.1	37.8	50.0	
Want to spend more time on direct patient contact (%)				0.030
Yes	52.9	81.1	75.0	
No	47.1	18.9	25.0	
Completely satisfied with current time-utilization (%)				0.272
Yes	2.9	10.8	15.0	
No	97.1	89.2	85.0	
Extra financial support from healthcare insurer to stimulate cooperation with GP (%)				0.226
Yes	26.5	18.9	40.0	
No	73.5	81.1	60.0	

The results show a few characteristics that may explain the difference in the amount of time being spent on CPS. However none of these characteristics is significantly associated with a different pattern of time utilization. Pharmacists that spend more time on CPS tend to consist more of locum pharmacists. In The Netherlands, when a locum pharmacist is present, there is always a resident pharmacist working in the pharmacy. So this could be the effect of task delegation, where the resident pharmacist focuses primarily on activities concerning pharmacy management and the locum pharmacist focusing primarily on CPS. Also, the residential area of where the community pharmacy is located in tends to consist of an older population in group 3 and could therefore have an increased need for CPS. Also, group 2 and 3 consist of more female pharmacists compared to group 1. This could imply that female pharmacists tend to spend more time on CPS. However, this is effect is probably due to the relatively high influx of female pharmacists into community pharmacy in the past decades. Therefore this effect is probably more likely to be attributable to age and the type of pharmacist (most younger community pharmacists work as a locum pharmacist) instead of gender.

Compared to group 3, groups 1 and 2 contain more resident pharmacists that also (partially) own a community pharmacy. Resident pharmacists that own a pharmacy are more likely to be responsible for pharmacy management than locum pharmacists. This result underlines the hampering effect of managerial activities on the amount of time that can be spent on CPS.

It could be expected that pharmacists working in pharmacies belonging to a chain of pharmacies or a partnership would be able to spend more time on CPS, as pharmacy management may more often be organized from a head office. However, the results do not support this. So the limited amount of time being spent on CPS due to the hampering effect of other activities seems to be present through the entire community pharmacy market. This has also been found in earlier research, also in the United States, that showed that pharmacists employed by drug chains and independent pharmacists did not differ both regarding desired and actual time spent on CPS [20, 21].

It has been suggested earlier in international literature that community pharmacists experience a lack of confidence or fear of new responsibilities when trying to provide CPS [22]. This could also explain why pharmacists are hesitant to spend more time on CPS. This could also explain that relatively young locum pharmacists are spending more time on CPS due to the fact that their education focused more on the provision of CPS [23]. This in contrary to older (resident) pharmacists who’s education focused on (analytical) chemistry and compounding instead of pharmacotherapy and patient counselling.

The results from this study also show that utilizing centralized prescription processing (CPP) does not influence the amount of time being spent on CPS. In the Netherlands, many pharmacists apply CPP that implies outsourcing of the preparation of a drug order and labelling to a central fill pharmacy. One of the benefits being that time normally devoted to picking and labelling in the pharmacy, can be redirected to other activities (hence CPS). Reason for the absence of this result could be due to staff reductions after the introduction of CPP and therefore not using CPP as a tool to redirect available time to CPS.

Community pharmacists that state they have full control of their time utilization, spend as much time on CPS as community pharmacists who state they have only partial control. This could be an indication that pharmacists do not feel a need to spend more time on CPS. However, concurrently the majority of pharmacists stated to want to spent more time on direct patient contact (being part of CPS). This is in line with previous research that showed that community pharmacists want to spend more time on consultation and medication management and less time on activities concerning dispensing and business management [20, 21]. Notable result found in this study is that pharmacists belonging to group 1 are, next to spending less time on CPS, also less eager to spend more time on direct patient contact compared to pharmacists from group 2 and 3. This result implies that some community pharmacists within group 1 are consciously avoiding the provision of CPS.

Studies, both from inside and outside the Netherlands. also showed that pharmacists were positive about services such as CMR and discharge counselling, but were experiencing a lack of time, lack of sufficient supporting staff and insufficient reimbursement [24, 25].

The lack of focus on CPS that is predominantly present in group 1 can be detrimental. As policymakers and professional bodies are trying to redefine the role of community pharmacy practice in the Netherlands, to address societal needs and also ensuring a long-term future for the profession, community pharmacists such as those belonging to group 1 can hamper this process.

On the other hand this was only a minority of all participants and furthermore we may also need pharmacists who concentrate on ‘back-office’ tasks. As long as these pharmacists are joined with more CPS oriented pharmacists there may not be an issue.

### Strengths and limitations

The characteristics of the community pharmacists who participated in this study are largely comparable to known characteristics of all community pharmacists in The Netherlands: 33% male compared to 46% nationally, 66% resident pharmacists compared to 72% nationally, 0.97 full-time equivalent (FTE) versus 0.89 FTE nationally [26].

Using a smartphone application to gather work-sampling data is considered user-friendly and an efficient way to attract participants to generate more data. The self-reporting aspect limits the “Hawthorne” effect, as participants feel less scrutinized than when being observed. This will limit behavioral changes in this study [27]. In previous research, it has been found that sensitivity analysis showed no major differences between responses given within an hour versus responses after more than an hour [11]. It has also been stated earlier that pharmacists have better understanding of their (current) activities than observers would have [28].

However, this type of research methodology also comes with disadvantages. Participants may provide socially desirable answers in using both the smartphone application, but also when providing background information (e.g. wanting to spend more time on CPS). However, it is expected that this effect is limited due to the provision of insights into time utilization and benchmark data. Also, using a smartphone application meant that participants had to keep their phone with them as much as possible, which could be undesirable in daily practice. Thereby, only pharmacists with good and coordinated workstreams may have participated, because only they may have had the time to participate. This would generate recruitment bias. A total of 65 initial participants did not provide background information which could be a result of this. However, participating pharmacists were provided with information about their own time utilization. This might have encouraged pharmacists who are struggling with their time utilization to also participate [11]. Also, the group of 65 excluded participants contained participants who may not have had the intention to fully participate in the study. Within this group a relatively high proportion downloaded the smartphone application but only used it a limited number of times. They installed the application and registered a few activities, but dropped out early. Participating pharmacists were provided with information about their own time utilization, including a benchmark of other pharmacists. We expect that this will have encouraged pharmacists who are struggling with their time utilization to participate [11]. Moreover, feedback and benchmarking is likely to stimulate honest reporting.

## Conclusion

Time dedicated to the provision of CPS has to be balanced with time dedicated to pharmacy management, logistics and other activities. Community pharmacists spending more time on CPS, predominantly spend less time on pharmacy management. Pharmacists spending more time on CPS compared to others tend to be locum pharmacists or work at a community pharmacy located in a residential area with largely older inhabitants, however these characteristics were not very strong predictors, suggesting that there probably are additional characteristics of pharmacists or pharmacies that determine the time spent on CPS.

## Appendix

**Table:**

Composed activity group	Activity group from [10]	Sub activities belonging to activity group from [10]
Cognitive pharmaceutical services	Cognitive pharmaceutical services	Medication use review
		Care-related contact with a patient (not a medication review)
		Contact with another healthcare professional(s) about a patient (not a medication review)
		Periodic meeting with GPs and pharmacists on prescribing policy
		Updating clinical information from patients
		Clinical rules
Pharmacy management	Organizational activities	Contact with other healthcare professionals for organizational reasons
		Preparing or attending work meetings
		General organization management
	Human resource management	General employee management
		One-on-one conversation with an employee, e.g. job performance evaluation
		Supervising an intern
		Hiring new employees
		Making and updating work schedules
		Employee administration, e.g. salary, worked hours etc.
		General employee management
	Finance	Assessing contracts with health insurance companies
		Checking declarations and authorizations
		Administrative tasks for patients
		Registering cash money in cash register and/or safe
		General financial administration
Quality assurance	Quality assurance	Supervising an audit
		Working on the quality manual
		General quality assurance management
		Investigating customer satisfaction
		Investigating satisfaction with other healthcare professionals
	Final check of prescription	Checking for inappropriate prescribing and possible distribution errors
	Clinical risk management	Checking for inappropriate prescribing and faulty drug combinations
Logistical processes	Logistics	Contact with a patient about logistics
		Stock management
		Processing a recall
		Stock-taking
		Processing orders from the wholesaler
	Dispensing process	Processing prescriptions
		Filling prescriptions
		Checking filled prescriptions
		Hand out filled prescriptions to patients
		Preparing or checking a prepared drug
		Copying prescriptions for the digital archive
Other	Household chores	General housekeeping tasks
	Education	Following a refresher course
		Teaching a refresher course to others
		Studying work-related literature
	Non-professional encounters and other	Conducting research for other institutions
		Processing mail and e-mail
		General chat with other healthcare professional(s)
		General chat with a patient
	Rest	Taking a break at work
